# Quantum algorithm for electronic band structures with local tight-binding orbitals

**DOI:** 10.1038/s41598-022-13627-x

**Published:** 2022-06-14

**Authors:** Kyle Sherbert, Anooja Jayaraj, Marco Buongiorno Nardelli

**Affiliations:** Department of Physics, University of North Texas, Denton, TX 76203 USA

**Keywords:** Electronic structure, Qubits, Information theory and computation

## Abstract

While the main thrust of quantum computing research in materials science is to accurately measure the classically intractable electron correlation effects due to Coulomb repulsion, designing optimal quantum algorithms for simpler problems with well-understood solutions is a useful tactic to advance our quantum “toolbox”. With this in mind, we consider the quantum calculation of a periodic system’s single-electron band structure over a path through reciprocal space. Previous efforts have used the Variational Quantum Eigensolver algorithm to solve the energy of each band, which involves numerically optimizing the parameters of a variational quantum circuit to minimize a cost function, constructed as the expectation value of a Hamiltonian operator. Traditionally, a unique Hamiltonian operator is constructed for each k-point, so that many cost functions, each with their own parameter space, must be optimized to generate a single band. Similarly, calculating higher bands than the first has traditionally involved modifying the cost function with additional overlap terms to ensure higher-energy eigenstates are orthogonal to those of lower bands. In this paper, we adopt a direct space approach, using a novel hybrid first/second-quantized qubit mapping which allows us to construct a single Hamiltonian, and a single cost-function, suitable for solving the entire electronic band structure. In contrast to previous approaches, the k-point and the band index are selected by additional parameters in our quantum circuit, rather than through modifications to the cost function. The result is a technically and conceptually simpler approach to band structure calculations on a quantum computer. Moreover, we expect that the tools developed herein will motivate new strategies for tackling highly-correlated materials beyond the grasp of classical computing.

## Introduction

Electronic band structures plot the energy eigenstates of an electron in the presence of a periodic potential as a function of momentum. They yield many useful properties of solid-state materials: for example, the presence of band gaps is one prerequisite of designing a semi-conductor, and the shape of a band informs electronic transport properties. Band structures are easily calculated classically under the single-electron approximation, but extending the approach to highly correlated materials has been challenging. This obstacle has motivated many materials scientists to consider quantum computation, which enables extensions to the the band structure approach which more accurately account for electron–electron interactions. The literature is full of distinct algorithms which treat periodic systems, including hybrid quantum-classical DMFT^1–4^, cyclic qubit arrays^5^, and “holographic” VQE^6^. Other methods developed for quantum chemistry are also readily applied to periodic systems expanded in a basis of plane waves^7^ or Bloch atomic orbitals^8–10^. We refer the reader to Bauer et al.^11^ for a thorough review of the most popular approaches.

Several recent papers^9,12,13^ have demonstrated electronic band structure calculations using the Variational Quantum Eigensolver (VQE) algorithm^14–16^. VQE is a popular quantum algorithm in the Noisy Intermediate-Scale Quantum (NISQ) era which consists of a low-depth parameterized quantum circuit preparing a variational ansatz $|\Psi (\theta )\rangle$, and a Hamiltonian operator ${\hat{H}}$ whose expectation value can be measured in the quantum computer. The variational parameters are adjusted until the energy $E \equiv \langle \Psi |{\hat{H}}|\Psi \rangle$ is minimized. This energy is a good approximation of the ground state of the system described by ${\hat{H}}$, provided that the ansatz is sufficiently expressive. Many ansatze have been developed for electronic structure calculations, balancing expressivity with efficient implementation on available quantum hardware^10,17–21^. In band structure calculations, the ansatz $|\Psi (\theta )\rangle$ is constrained to a single-electron wavefunction, and the ground state is the energy of the lowest band at a particular momentum $\mathbf{k}$. Higher bands are found by repeating the optimization with additional constraints to ensure the ansatz is orthogonal to previously located eigenstates.

In order to estimate the expectation value of the Hamiltonian ${\hat{H}}$ in a quantum computer, ${\hat{H}}$ should be expressed as a linear combination of “Pauli words”:

1 $$\begin{aligned} {\hat{H}} \rightarrow \sum _j c_j {\hat{P}}_j. \end{aligned}$$

Each Pauli word ${\hat{P}}_j=\{{\hat{I}}, {\hat{X}}, {\hat{Y}}, {\hat{Z}}\}^{\otimes n}$ is an *n*-qubit operator with a Pauli spin matrix associated with each qubit. The precise choice of $\{c_j\}$ depends on the choice of orbital basis, and how this basis is mapped onto the available logical qubits. Previous results for solving electronic band structures each used somewhat different strategies for the qubit mapping, but all three adopted a Bloch atomic orbital basis^9,12,13^. Each Bloch atomic orbital $|\alpha _\mathbf{k }\rangle$ is related to the local atomic orbitals $\{|\alpha _\mathbf{r }\rangle \}$ at each lattice point $\mathbf{r}$ in the crystal by a Fourier transformation:

2 $$\begin{aligned} |\alpha _\mathbf{k }\rangle = \sum _\mathbf{r} e^{i\mathbf{k} \cdot \mathbf{r} } |\alpha _\mathbf{r }\rangle . \end{aligned}$$

Bloch atomic orbitals form a valuable basis for band structure calculations because the basis states are intrinsically periodic, and any wavefunction constructed in this basis automatically satisfies the periodicity of the system. Furthermore, the single-electron Hamiltonian is separable in $\mathbf{k}$, meaning that each set of orbitals $|\alpha _\mathbf{k }\rangle$ for fixed $\mathbf{k}$ can be solved independently, reducing the effective size of the eigenvalue problem. However, this comes with the consequence that a new set of $\{c_j\}$ must be obtained for every $\mathbf{k}$. Similarly, while previous results used different strategies for exploring higher-level bands, they each enforced orthogonality with previously located eigenstates by including additional terms in the cost function. This effectively generates a new set of $\{c_j\}$ for each *band*, and often increases the number of quantum measurements required to obtain each *E*.

In this paper, we present an algorithm which simplifies electronic band structure calculations in a quantum computer by setting the cost function just once, for all $\mathbf{k}$ and for all bands. We accomplish this by adopting the basis of local atomic orbitals, and by enforcing the periodicity of our eigenstates directly through the ansatz. In this framework, $\mathbf{k}$ enters as an additional (fixed) parameter to our variational quantum circuit. Additionally, we present a novel procedure for exploring higher bands without the use of additional terms in the cost function. Instead, orthogonality with previously located eigenstates is enforced by the quantum circuit itself, incurring negligible overhead in circuit complexity and no overhead in the required number of measurements.

The “Qubit mapping, “Quantum circuit”, and “Cost function” sections present the technical details of our algorithm. The “Examples” section briefly presents examples using simulated results to validate our method. In the “Conclusion”, we discuss the advantages and disadvantages of our approach, and we suggest how this method could be adapted to efficiently treat highly-correlated systems.

## Qubit mapping

In this paper, we adopt a local atomic orbital basis. We associate with each unit cell a lattice coordinate $\mathbf{r}$ and a set of *M* orbitals $\{|\alpha \rangle \}$, such as the hydrogen-like orbitals $|s\rangle$, $|p_x\rangle$, etc. centered on each atom. The set $\{|\alpha _\mathbf{r }\rangle \}$ of all orbitals over all unit cells forms the basis for the crystal. In practice, we restrict ourselves to a supercell consisting of *N* total unit cells. The value of *N* determines the resolution in $\mathbf{k}$ we can obtain in our band structure, where the limit $N\rightarrow \infty$ corresponds to a continuous $\mathbf{k}$ space. We index each unit cell with an integer coordinate $\nu$ counting from $\mathbf{0}$ to *N*, thereby adopting the finite basis $\{|\alpha _{\nu }\rangle \}$ with *MN* elements. We use the bold font to indicate that in a *d*-dimensional crystal, $\nu$ takes the form of a *d*-tuple.

We map our supercell onto a set of qubits using a novel hybrid first/second-quantized approach, factoring the basis orbital $|\alpha _{\nu }\rangle$ into two sub-states:

3 $$\begin{aligned} |\alpha _{\nu }\rangle = |\alpha \rangle \otimes |\nu \rangle . \end{aligned}$$

**Figure 1:**
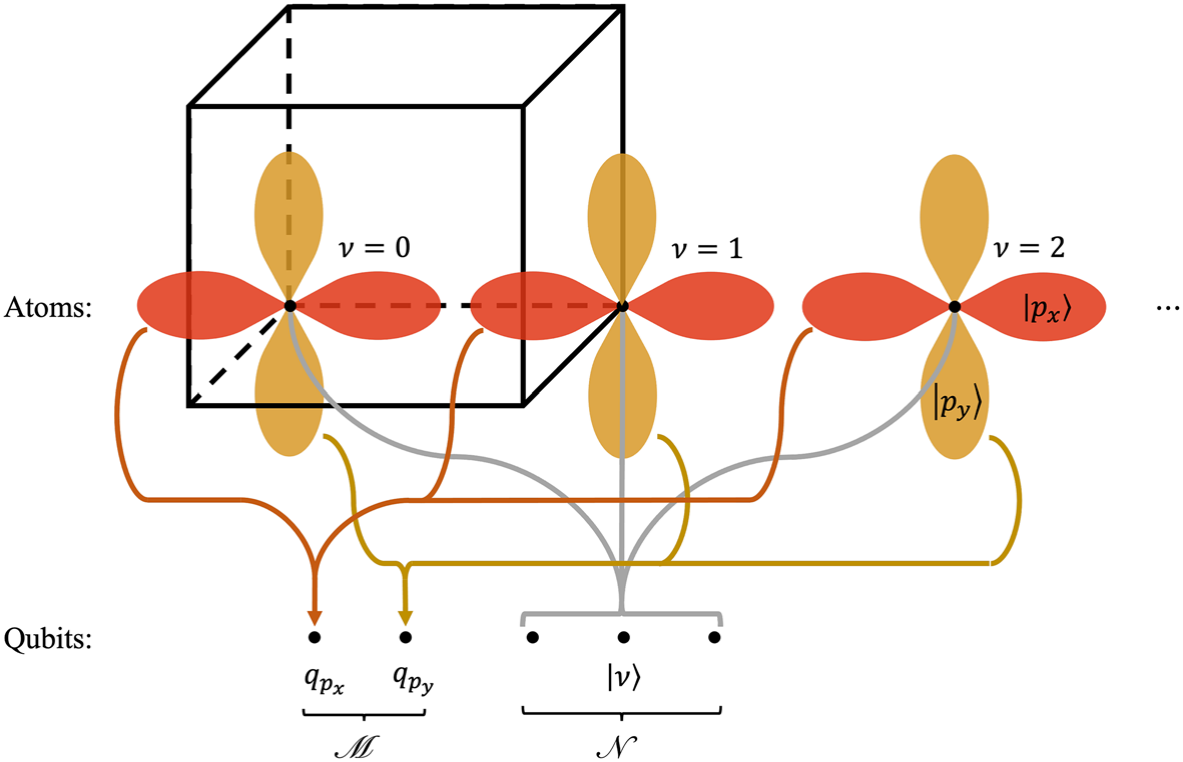
A schematic illustrating the hybrid qubit mapping used in this paper. Each unit cell in a supercell is indexed with the “site register” using first quantization. Meanwhile, each qubit in the “orbital register” is associated with a single orbital in each unit cell using second quantization. For example, in this schematic, the qubit labelled $q_{p_x}$ holds the amplitude for every $p_x$ orbital in the supercell, while the site register contributes the phase for each site.

The $|\alpha \rangle$ state is mapped onto an “orbital register” $\mathscr {M}$ consisting of *M* qubits encoded with second quantization. Each local atomic orbital $\alpha$ is associated with a specific qubit $q_\alpha \in \mathscr {M}$; the state $|\alpha \rangle$ corresponds to the computational basis state in which $q_\alpha$ is $|1\rangle$ and all other qubits are $|0\rangle$. The $|\nu \rangle$ state is mapped onto a “site register” $\mathscr {N}$ consisting of $\log N$ qubits encoded with first quantization. Each basis state of $\mathscr {N}$ is the binary representation of the integer coordinate $\nu$; a *d*-dimensional crystal will have *d* sub-registers in $\mathscr {N}$. The total number of qubits required by this mapping is $M+\log N$. Fig. 1 shows a schematic of our qubit mapping.

## Quantum circuit

A general ansatz expanded in the local atomic orbital basis $\{|\alpha _\mathbf{r }\rangle \}$ may be written as

4 $$\begin{aligned} |\Phi \rangle = \sum _{\alpha } \sum _\mathbf{r } \phi _{\alpha \mathbf{r} } |\alpha _\mathbf{r }\rangle . \end{aligned}$$

Bloch’s theorem guarantees that single-electron eigenstates of a periodic system must themselves be periodic, with a phase factor determined by the momentum of the electron $\mathbf{k}$:

5 $$\begin{aligned} \phi _{\alpha \mathbf{r} } = e^{i\mathbf{k} \cdot \mathbf{r} } \phi _{\alpha \mathbf{0} }. \end{aligned}$$

For ease of notation, let us assume a one-dimensional crystal with lattice constant *a*, so that each lattice coordinate $\mathbf{r} \rightarrow a\nu$ for an integer coordinate $\nu$. Imposing periodic boundary conditions on a supercell of size *N* requires $\phi _{\alpha ,aN}=\phi _{\alpha ,0}$, implying

6 $$\begin{aligned} ka = \frac{2\pi }{N} p \end{aligned}$$

for a momentum quantum number *p*.

Under the qubit mapping described above, we can combine Eqs. (3), (4), (5), and (6):

7 $$\begin{aligned} |\Phi \rangle = \bigg ( \sum _{\alpha } \phi _{\alpha } |\alpha \rangle \bigg )_\mathscr {M}\otimes \bigg ( \frac{1}{\sqrt{N}} \sum _{\nu } e^{\frac{2\pi i}{N} p \nu } |\nu \rangle \bigg )_\mathscr {N}. \end{aligned}$$

The first factor describes the amplitudes of each orbital on the principal unit cell and is expressed in the orbital register $\mathscr {M}$, while the second factor describes the phases accumulated on each site and is expressed in the site register $\mathscr {N}$. We have absorbed a factor of $\sqrt{N}$ from the site register into the amplitudes $\phi _{\alpha } \equiv \sqrt{N} \phi _{\alpha ,0}$ so that both registers are normalized. The action on both registers is factorized and thus can be expressed with two independent quantum circuits.

### Site register

We wish to prepare the following state in the site register $\mathscr {N}$, from the starting state $|\mathbf{0} \rangle$

8 $$\begin{aligned} |\Phi \rangle _\mathscr {N}= \frac{1}{\sqrt{N}} \sum _{\nu } e^{\frac{2\pi i}{N} p \nu } |\nu \rangle . \end{aligned}$$

**Figure 2:**
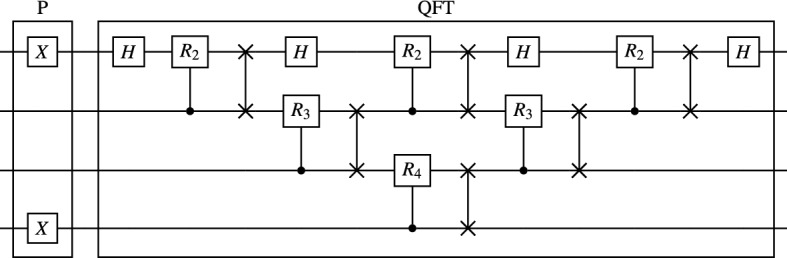
The quantum circuit applied on our site register $\mathscr {N}$. The sub-circuit P loads the computational basis state $|p\rangle$. The sub-circuit QFT implements the Quantum Fourier Transform on a linear architecture.

This state is determined exactly by the momentum quantum number *p*, requiring no other variational parameters. Equation (8) has the form of a discrete Fourier transform, and we can make use of the well-known Quantum Fourier Transform (QFT), an efficient implementation of the discrete Fourier transform as a quantum circuit^22^. We first prepare the state $|p\rangle$ with a single layer of Pauli *X* gates applied to each bit for which the binary representation of *p* is $|1\rangle$. We then apply QFT, preparing the state in Eq. (8) exactly. Fig. 2 presents an implementation of QFT optimized for linear qubit architectures, requiring $\Theta ((\log N)^2)$ entangling gates and $\Theta (\log N)$ depth.

### Orbital register

We wish to prepare the following state in the orbital register $\mathscr {M}$, from the starting state $|\mathbf{0} \rangle$:

9 $$\begin{aligned} |\Phi \rangle _\mathscr {M}= \sum _{\alpha } \phi _{\alpha } |\alpha \rangle . \end{aligned}$$

**Figure 3:**
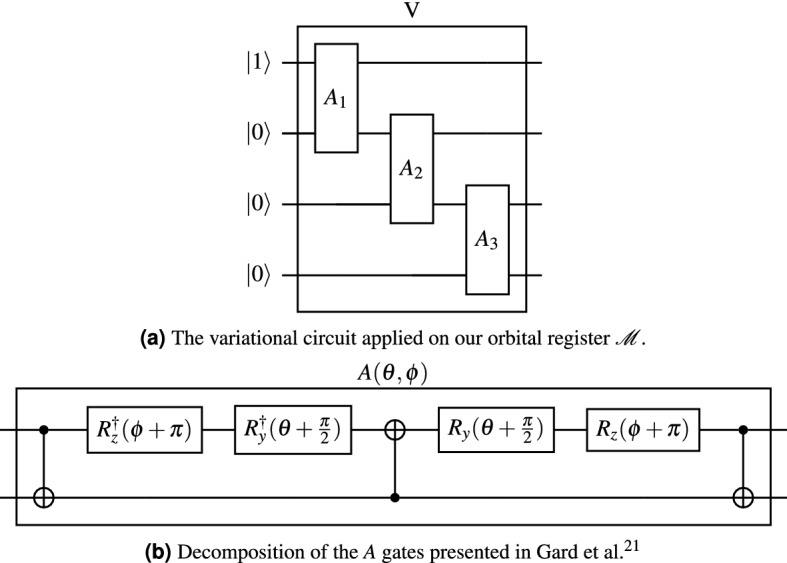
(**a**) Shows the variational quantum circuit applied on our orbital register $\mathscr {M}$ to locate the lowest band of any periodic system. (**b**) Decomposes the *A* gate into elementary circuit elements, each consisting of two independent parameters.

The amplitudes $\phi _{\alpha }$ are *a priori* unknown, and we will treat them as variational parameters. Our objective is to design a parameterized quantum circuit capable of expressing any arbitrary superposition of the basis states $|\alpha \rangle$, which are those *M*-qubit computational basis states with a Hamming weight of 1. The circuit *V* presented in our previously-published work^13^ and re-presented in Fig. 3a is suitable for locating the lowest band. It consists of $M-1$ so-called *A* gates (Fig. 3b), first presented in Gard et al.^21^ each of which accepts a polar angle $\theta$ and an azimuthal angle $\varphi$, for a total of $2(M-1)$ independent parameters. If desired, the ansatz may be constrained to real values by setting all azimuthal angles to 0.

**Figure 4:**
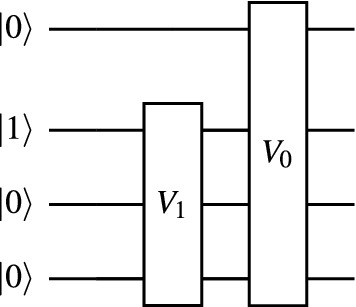
The variational circuit applied on our orbital register $\mathscr {M}$ to locate the second-lowest band of any periodic system. The $V_0$ sub-circuit is as shown in Fig. 3a. The $V_1$ circuit is analogously constructed, save with one fewer qubit.

We now wish to consider how to adapt *V* for higher bands, in a way which ensures orthogonality with previously located eigenstates. Let us define $V_0$ as that circuit *V* with parameters optimized to prepare the eigenstate $|\psi _0\rangle$ of the lowest band, so that $V_0 |10..0\rangle = |\psi _0\rangle$. If $V_0$ is applied to any other computational basis state, the result must be orthogonal to $|\psi _0\rangle$. Therefore, if we prepare an arbitrary single-electron state $|\Psi \rangle$ over all qubits but the first (ie. $|\Psi \rangle =|0\rangle \otimes |\Psi _+\rangle$ for an arbitrary single-electron state $|\Psi _+\rangle$ with $M-1$ orbitals), the state $V_0|\Psi \rangle$ will be orthogonal to the ground-state $|\psi _0\rangle$ (Fig. 4). Thus, we may use the same variational form *V* for each band *l*, except that we omit qubit $q_l$ after each iteration, and we apply each previously optimized circuit $V_l$.

**Figure 5:**
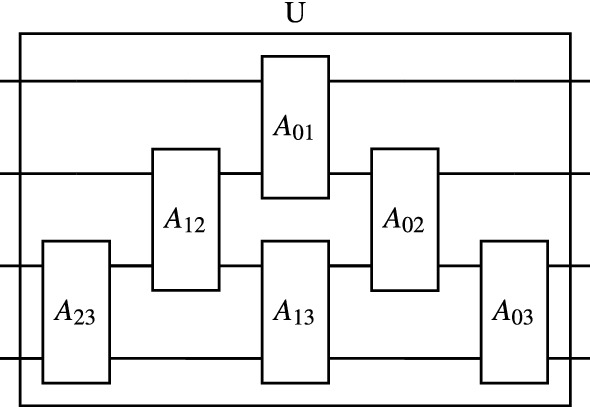
The generalized variational quantum circuit applied on our orbital register $\mathscr {M}$ to calculate any band of a periodic system. Each diagonal corresponds to a particular energy level. Parameters for bands lower than the target energy level are fixed at their optimal values, while parameter for bands higher than the target energy level are fixed at zero. Thus, only the parameters for the target band must be optimized.

The entire circuit for the orbital register can be compactly represented as in Fig. 5. It consists of $(M-1)(M-2)/2$ *A* gates, for a total of $\Theta (M^2)$ parameters. However, each optimization varies only those $M-(l+1)$ $A^{(l)}_q$ gates for which *l* is the index of the band currently being solved. The parameters of all lower *l* are fixed at the values which optimized band *l*, and the parameters of all higher *l* are fixed at 0. This circuit requires $O(M^2)$ entangling gates and $\Theta (M)$ depth.

## Cost function

Our objective in this section is to present a cost-function suitable for electronic band structure calculations over all $\mathbf{k}$ and all bands. We first write the tight-binding Hamiltonian ${\hat{H}}$ in the basis of local atomic orbitals $|\alpha _{\nu }\rangle$ described above, and map it onto a set of Pauli words so that ${\hat{H}} \rightarrow \sum _j c_j {\hat{P}}_j$. Ostensibly, the cost-function is $E = \langle {{\hat{H}}}\rangle = \sum _j c_j \langle {{\hat{P}}}\rangle$, where each $\langle {{\hat{P}}\rangle _j}$ can be estimated independently in a quantum computer^15^. However, we will also present a measurement strategy which ensures that the number of circuit measurements required to evaluate *E* scales minimally with the number of commuting groups in ${\hat{H}}$.

### Hamiltonian mapping

The single-electron tight-binding Hamiltonian ${\hat{H}}$ is expanded in our basis as follows:

10 $$\begin{aligned} {\hat{H}} = \sum _{\alpha ,\beta } \sum _{\nu ,\nu '} \langle \alpha _{\nu }|{\hat{H}}|\beta _{\nu '}\rangle \left (|\alpha \rangle \langle \beta |_\mathscr {M}\otimes |\nu \rangle \langle \nu '|_\mathscr {N}\right ). \end{aligned}$$

The physics of ${\hat{H}}$ is contained entirely within the real-valued hopping parameters $t_{\alpha \beta }^{(\delta )} \equiv \langle \alpha _\mathbf{0 }|{\hat{H}}|\beta _{\delta }\rangle$, which denote the energy cost of an electron transitioning from the orbital $|\beta _{\delta }\rangle$ to the orbital $|\alpha _\mathbf{0 }\rangle$ in the principal unit cell. The periodicity of the crystal ensures that all matrix elements can be written in terms of the hopping parameters:

11 $$\begin{aligned} \langle \alpha _{\nu }|{\hat{H}}|\beta _{\nu '}\rangle = t_{\alpha \beta }^{\bigg (\nu '-\nu \bigg )}. \end{aligned}$$

Note that hopping parameters between orbitals centered on atoms far apart (ie. large $\delta$) will tend to vanish. Thus, one typically adopts a “nearest-neighbor approximation”, in which $t_{\alpha \beta }^{(\delta )}$ is non-zero *only* for those unit-cells $\delta$ which hold the nearest images of $|\beta \rangle$ to the atomic center of $|\alpha _\mathbf{0 }\rangle$. Realness and hermiticity of ${\hat{H}}$ guarantee $t_{\alpha \beta }^{(\delta )} = t_{\beta \alpha }^{(-\delta )}$. The number of independent hopping parameters can often be reduced further by exploiting additional crystal symmetries, but we take all hopping parameters as given for the purposes of this work.

The orbital register projection $|\alpha \rangle \langle \beta |$ can be written as the annihilation of an electron in orbital $|\beta \rangle$ followed by the creation of an electron in orbital $|\alpha \rangle$, encouraging us to use the fermionic creation and annihilation operators $a^{\dagger }_{\alpha }$, $a_{\alpha }$:

12 $$\begin{aligned} |\alpha \rangle \langle \beta | = a^\dagger _\alpha a_\beta . \end{aligned}$$

The fermionic creation and annihilation operators are typically mapped onto Pauli words using well-known transformations such as the Jordan–Wigner or Bravyi–Kitaev transformations, which enforce the fermionic anticommutation relations necessary for representing indistinguishable electrons^23^. However, as pointed out in our previous work^13^, this full machinery is unnecessary for band structure calculations constrained to single-electron states, and the following, simpler mappings suffice:

13a $$\begin{aligned} a _\alpha&\rightarrow \frac{1}{2}( {\hat{X}}_\alpha + i {\hat{Y}}_\alpha ); \end{aligned}$$

13b $$\begin{aligned} a^\dagger _\alpha&\rightarrow \frac{1}{2}( {\hat{X}}_\alpha - i {\hat{Y}}_\alpha ), \end{aligned}$$

where we have used the notation ${\hat{X}}_\alpha$ (${\hat{Y}}_\alpha$) to represent the Pauli word with an ${\hat{X}}$ (${\hat{Y}}$) on the qubit $q_\alpha$ and the identity ${\hat{I}}$ on every other qubit.

The site register projection $|\nu \rangle \langle \nu '|$ must bring the basis state $|\nu '\rangle$ into the state $|\nu \rangle$, and it must annihilate every other state. This action is factorizable into each qubit:

14 $$\begin{aligned} |\nu \rangle \langle \nu '| = \bigotimes _q |\nu _q\rangle \langle \nu _q'|, \end{aligned}$$

where $\nu _q$ represents the *q*-th bit in the binary representation of the integer coordinate $\nu$. The reader may verify the following mappings for $|\nu _q\rangle \langle \nu _q'|$ hold:

15a $$\begin{aligned} |0\rangle \langle 0|&\rightarrow \frac{1}{2}( {\hat{I}} + {\hat{Z}} ); \end{aligned}$$

15b $$\begin{aligned} |0\rangle \langle 1|&\rightarrow \frac{1}{2}( {\hat{X}} + i {\hat{Y}} ); \end{aligned}$$

15c $$\begin{aligned} |1\rangle \langle 0|&\rightarrow \frac{1}{2}( {\hat{X}} - i {\hat{Y}} ); \end{aligned}$$

15d $$\begin{aligned} |1\rangle \langle 1|&\rightarrow \frac{1}{2}( {\hat{I}} - {\hat{Z}} ). \end{aligned}$$

More concisely,

16 $$\begin{aligned} |\nu _q\rangle \langle \nu _q'| \rightarrow \frac{1}{2} \bigg ({\hat{X}}^{\nu _q'}\bigg ) \bigg ({\hat{X}}^{\nu _q \oplus \nu _q'}\bigg ) ({\hat{I}} + {\hat{Z}}) \bigg ({\hat{X}}^{\nu _q'}\bigg ). \end{aligned}$$

(Note the use of the $\oplus$ operator to denote bitwise addition, also known as the “XOR” operator).

Given all hopping parameters $t_{\alpha \beta }^{(\delta )}$, Eqs. (10)–(16) are sufficient to generate the mapping ${\hat{H}} \rightarrow \sum _j c_j {\hat{P}}_j$. Generally, ${\hat{H}}$ will consist of $O(M^2 N^2)$ non-zero Pauli words. Adopting a nearest-neighbor approximation reduces this to $O(M^2 N)$. In the next section, we will show how these Pauli words can be partitioned into $O(M \log N)$ commuting groups to generate a cost function $E=\langle {{\hat{H}}}\rangle$ which can be efficiently evaluated in a quantum computer.

### Measurement strategy

We now write out the full single-electron tight-binding Hamiltonian under a nearest-neighbor approximation. The methods below generalize to multiple dimensions and are easily implemented in code, but the equations become very unwieldy, so we will restrict ourselves in this section to the one-dimensional case. Under the nearest-neighbor approximation, $t_{\alpha \beta }^{(\delta )}$ will be non-zero only if $\delta \in \{0, \pm 1\}$, and we can expand the Hamiltonian ${\hat{H}}$ from above as

17 $$\begin{aligned} {\hat{H}} = {\hat{H}}^{(0)} + {\hat{H}}^{(+1)} + {\hat{H}}^{(-1)}, \end{aligned}$$

where each ${\hat{H}}^{(\delta )}$ is defined as follows:

18a $$\begin{aligned} {\hat{H}}^{(0)}&\equiv \bigg (\sum _{\alpha ,\beta } t_{\alpha \beta }^{(0)} a^\dagger _\alpha a_\beta \bigg )_\mathscr {M}\otimes \bigg (\sum _{\nu } |\nu \rangle \langle \nu |\bigg )_\mathscr {N}; \end{aligned}$$

18b $$\begin{aligned} {\hat{H}}^{(+1)}&\equiv \bigg (\sum _{\alpha ,\beta } t_{\alpha \beta }^{(+1)} a^\dagger _\alpha a_\beta \bigg )_\mathscr {M}\otimes \bigg (\sum _{\nu } |\nu \rangle \langle \nu +1|\bigg )_\mathscr {N}; \end{aligned}$$

18c $$\begin{aligned} {\hat{H}}^{(-1)}&\equiv \bigg (\sum _{\alpha ,\beta } t_{\alpha \beta }^{(-1)} a^\dagger _\alpha a_\beta \bigg )_\mathscr {M}\otimes \bigg (\sum _{\nu } |\nu +1\rangle \langle \nu |\bigg )_\mathscr {N}. \end{aligned}$$

Note that we impose periodic boundary conditions so that the projection $|\nu \rangle \langle \nu +1|$ for $\nu =N-1$ is identified with $|N-1\rangle \langle 0|$.

Consider first the orbital register factors $\sum _{\alpha ,\beta } t_{\alpha \beta }^{(\delta )} a^\dagger _\alpha a_\beta$. Substituting Eq. (13),

19a $$\begin{aligned} \sum _{\alpha ,\beta }&t_{\alpha \beta }^{(\delta )} a^\dagger _\alpha a_\beta \equiv {\hat{A}}^{(\delta )}_\mathscr {M}+ i {\hat{B}}^{(\delta )}_\mathscr {M}; \end{aligned}$$

19b $$\begin{aligned} {\hat{A}}^{(\delta )}_\mathscr {M}=&\frac{1}{2} \sum _\alpha t_{\alpha \alpha }^{(\delta )} ({\hat{I}} - {\hat{Z}}_\alpha ) \nonumber \\&+ \frac{1}{4} \sum _\alpha \sum _{\beta >\alpha } \bigg (t_{\beta \alpha }^{(\delta )} + t_{\alpha \beta }^{(\delta )}\bigg ) ({\hat{X}}_\alpha {\hat{X}}_\beta + {\hat{Y}}_\alpha {\hat{Y}}_\beta ); \end{aligned}$$

19c $$\begin{aligned} {\hat{B}}^{(\delta )}_\mathscr {M}=&\frac{1}{4} \sum _\alpha \sum _{\beta >\alpha } \bigg (t_{\beta \alpha }^{(\delta )} - t_{\alpha \beta }^{(\delta )}\bigg ) ({\hat{Y}}_\alpha {\hat{X}}_\beta - {\hat{X}}_\alpha {\hat{Y}}_\beta ). \end{aligned}$$

In the real part ${\hat{A}}^{(\delta )}_\mathscr {M}$, all terms of the forms ${\hat{Z}}_\alpha$, ${\hat{X}}_\alpha {\hat{X}}_\beta$, and ${\hat{Y}}_\alpha {\hat{Y}}_\beta$ are each commutative, so that ${\hat{A}}^{(\delta )}_\mathscr {M}$ contains three commuting Pauli groups. In the imaginary part ${\hat{B}}^{(\delta )}_\mathscr {M}$, all terms of the form ${\hat{Y}}_\alpha {\hat{X}}_{\beta >\alpha }$ and ${\hat{X}}_\alpha {\hat{Y}}_{\beta >\alpha }$ are each commutative for fixed $\alpha$, so that ${\hat{B}}^{(\delta )}_\mathscr {M}$ contains $\Theta (M)$ Pauli groups.

Consider now the site register factors in Eq. (18). The sum $\sum _\nu |\nu \rangle \langle \nu |$ is immediately recognized as the identity operator ${{\hat{\mathscr {I}}}_{\mathscr {N}}}$ acting on the site register. The sum $\sum _\nu |\nu \rangle \langle \nu +1|$ is less trivial, but it too can be decomposed into real and imaginary parts ${\hat{A}}_\mathscr {N}$, ${\hat{B}}_\mathscr {N}$:

20 $$\begin{aligned} \sum _\nu |\nu \rangle \langle \nu +1| \equiv {\hat{A}}_\mathscr {N}+ i {\hat{B}}_\mathscr {N}. \end{aligned}$$

In the Supplementary Information, we show that ${\hat{A}}_\mathscr {N}$ and ${\hat{B}}_\mathscr {N}$ each consist of $\Theta (\log N)$ commuting groups.

We now rewrite the Hamiltonian ${\hat{H}}$ of Eq. (17) in terms of the ${\hat{A}}, {\hat{B}}$ operators:

21 $$\begin{aligned} {\hat{H}} = {\hat{A}}^{(0)}_\mathscr {M}\otimes {{{\hat{\mathscr {I}}}}_{\mathscr {N}}} + 2 \bigg ( {\hat{A}}^{(1)}_\mathscr {M}\otimes {\hat{A}}_\mathscr {N}- {\hat{B}}^{(1)}_\mathscr {M}\otimes {\hat{B}}_\mathscr {N}\bigg ). \end{aligned}$$

We have used the symmetry relation $t_{\alpha \beta }^{(\delta )} = t_{\beta \alpha }^{(-\delta )}$ to find ${\hat{B}}^{(0)}_\mathscr {M}=0$. The same relation also ensures ${\hat{H}}^{(+1)}$ and ${\hat{H}}^{(-1)}$ are Hermitian conjugates, and we have replaced the sum ${\hat{H}}^{(+1)}+{\hat{H}}^{(-1)}$ by $2 \Re \left [{\hat{H}}^{(+1)}\right]$. The number of commuting groups in ${\hat{H}}$ is bounded by the $B^{(1)}_\mathscr {M}\otimes B_\mathscr {N}$ term, for a total of $\Theta (M \log N)$ commuting groups.

The main result of this section is the cost function $E=\langle {H}\rangle$, suitable for all $\mathbf{k}$ and for all bands:

22 $$\begin{aligned} {\hat{E}} = \bigg \langle {{\hat{A}}^{(0)}_\mathscr {M}\bigg \rangle } + 2 \bigg ( \bigg \langle {{\hat{A}}^{(1)}_\mathscr {M}\otimes {\hat{A}}_\mathscr {N}\bigg \rangle } - \bigg \langle {{\hat{B}}^{(1)}_\mathscr {M}\otimes {\hat{B}}_\mathscr {N}\bigg \rangle } \bigg ). \end{aligned}$$

**Figure 6:**
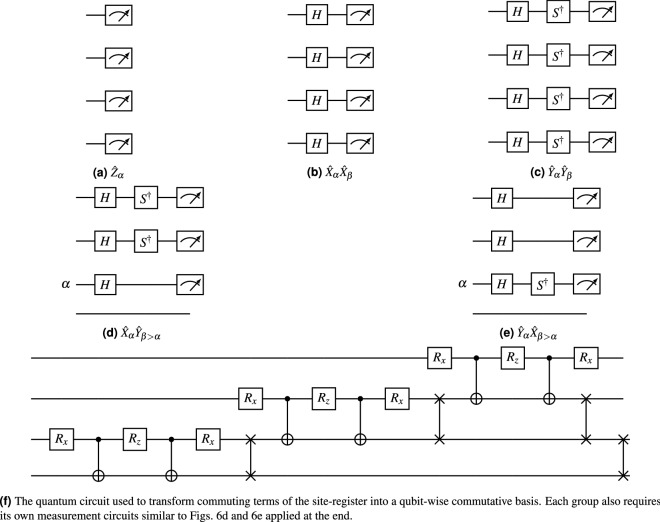
Measurement circuits to efficiently group all terms of the cost-function into commuting groups. (**a**–**e**) are each applied to the orbital register in subsequent simulations, while (**f**) is applied to the site register.

Each expectation value in *E* is measured by the quantum computer. Let $\mathscr {Q}_n$ be the set of all *n*-qubit Pauli words consisting of only ${\hat{I}}$ and ${\hat{Z}}$ operators. The procedure for simultaneously obtaining the expectation values $\langle {{\hat{Q}}_j}\rangle$ of all Pauli words ${\hat{Q}}_j \in \mathscr {Q}_n$ is well understood (see eg. our previous work^13^ for a brief tutorial). The same procedure can be applied to any commuting group $\mathscr {P}_n$ if a basis rotation circuit is applied prior to measurement which transforms each Pauli word ${\hat{P}}_j \in \mathscr {P}_n$ to an element of $\mathscr {Q}_n$^24^. For example, all Pauli words of the form ${\hat{X}}_\alpha {\hat{X}}_\beta$ in ${\hat{A}}^{(\delta )}_\mathscr {M}$ can be measured simultaneously by applying the Hadamard gate to each qubit prior to measurement. Figure 6 presents the measurement circuits required for each register to measure all of the commuting groups in ${\hat{H}}$.

So far, the measurement strategy discussed in this section is agnostic of the quantum circuit presented above. Our quantum circuit treats the orbital and site registers as independent, introducing no entanglement between the two. As a consequence, any expectation value $\langle {{\hat{O}}_\mathscr {M}{\hat{O}}_\mathscr {N}\rangle }=\langle {{\hat{O}}_\mathscr {M}\rangle }\cdot \langle {{\hat{O}}_\mathscr {N}\rangle }$, and we can estimate the expectation values of each ${\hat{A}}$ and ${\hat{B}}$ operator independently, reducing the number of circuit measurements to $\Theta (M+\log N)$. Going a step further, we see from Eq. (8) that $\langle {{\hat{A}}_\mathscr {N}\rangle }$ and $\langle {{\hat{B}}_\mathscr {N}\rangle }$ depend only on the momentum quantum number *p*, and we can evaluate them *a priori* as the real and imaginary parts of $\sum _\nu \langle \Phi _\mathscr {N}\vert \nu \rangle \langle \nu +1\vert \Phi _\mathscr {N}\rangle$:

23a $$\begin{aligned} \langle {{\hat{A}}_\mathscr {N}\rangle }&= \cos (\frac{2\pi }{N} p); \end{aligned}$$

23b $$\begin{aligned} \langle {{\hat{B}}_\mathscr {N}\rangle }&= \sin (\frac{2\pi }{N} p). \end{aligned}$$

Now we may write our cost-function as

24 $$\begin{aligned} E = \langle {{\hat{A}}^{(0)}_\mathscr {M}\rangle } + 2 \left[ \langle {{\hat{A}}^{(1)}_\mathscr {M}\rangle } \cdot \cos (\frac{2\pi }{N} p) - \langle {{\hat{B}}^{(1)}_\mathscr {M}\rangle } \cdot \sin (\frac{2\pi }{N} p) \right] . \end{aligned}$$

In this perspective, $\mathbf{k}$ enters in (via *p*) as a classical parameter of the cost function rather than as an input into the quantum circuit, establishing the equivalence of this method and those of previous results^9,12,13^. Choosing between Eqs. (22) and (24) is a matter of preference and logistical convenience.

## Examples

**Figure 7:**
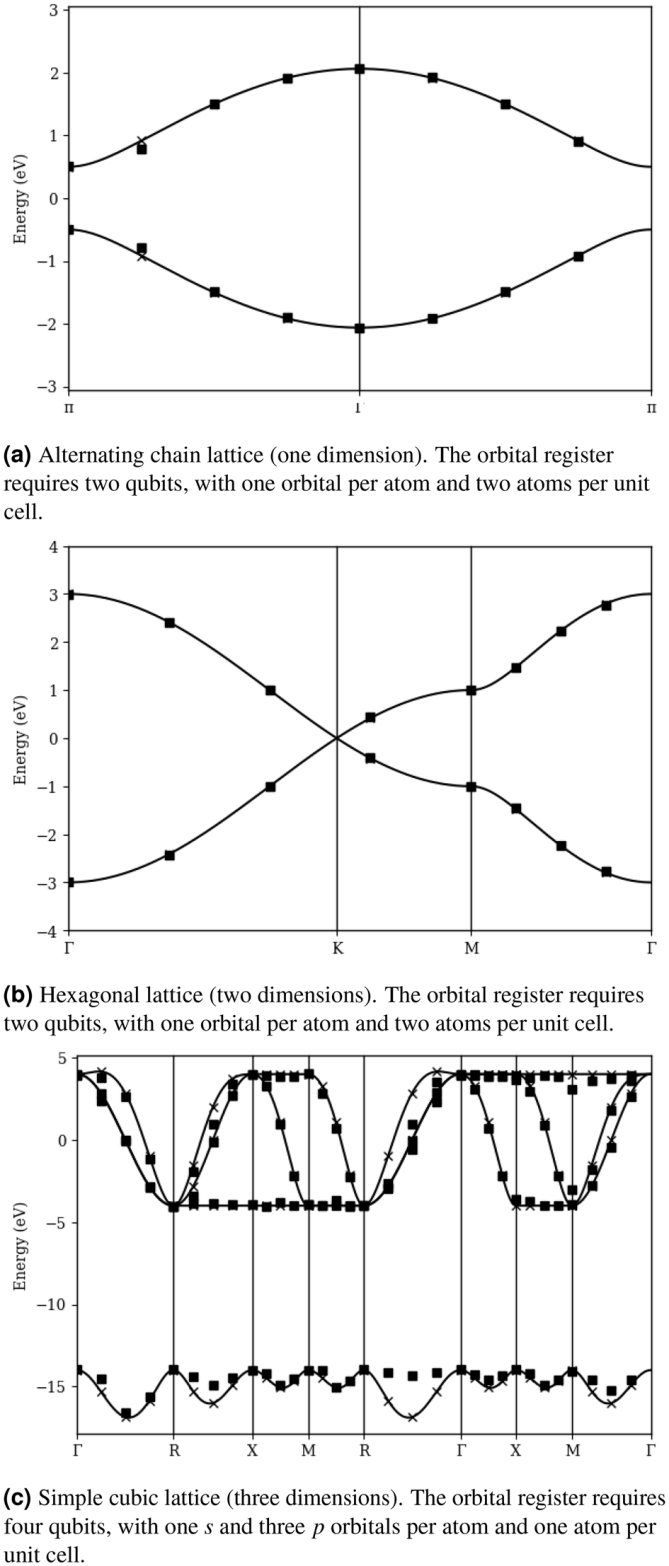
Band structures of model systems in one, two, and three dimensions, using $N=8$ for each dimension. The site register requires 3 qubits per dimension. Solid curves are calculated analytically with the standard classical algorithm. Squares mark the values estimated by simulating the quantum algorithm presented in this paper, using the COBYLA optimization protocol and 8096 circuit evaluations per expectation value. X’s mark the values obtained in ideal conditions, with perfect optimization and no sampling noise.

Figure 7 shows electronic band structures calculated for several model systems, using Eq. (22) and the circuits presented in Figs. 2, 3, 4, 5, 6. Figure 7a corresponds to a one-dimensional lattice consisting of alternating atoms. The unit cell consists of two distinct atoms, each contributing a single orbital with rest energies separated by 1 eV. The hopping parameter between each adjacent atom is also 1 eV. The lower band corresponds to the “bonding” orbital for individual electrons at each momentum, and the upper band corresponds to the “anti-bonding” orbital. The solid curve is calculated analytically with the standard classical algorithm. Our quantum solution requires two qubits for the orbital register, and we choose to use three qubits ($N=8$) for the site register, for a total of five qubits. (Contrast this with a direct simulation of all 16 atoms in the supercell, which requires sixteen qubits). Square markers are obtained by simulating our quantum circuits with Google’s cirq software package, using the COBYLA optimization protocol (as implemented in the scipy python package) and 8096 circuit evaluations per expectation value. When these results differ noticeably from the analytical solution, we use an X to denote the result of our algorithm in ideal conditions, with perfect optimization and no sampling noise.

Figure 7b corresponds to a two-dimensional hexagonal lattice, such as in graphene. The unit cell consists of two identical atoms, each contributing a single orbital. Each atom has three neighbors, each with a hopping parameter of 1 eV. Our results use a supercell size of $N=8$ for each dimension, requiring six qubits in the site register and two in the orbital register, for a total of eight qubits. One feature of special interest is the “Dirac cone” surrounding the degeneracy at the high-symmetry point labelled *K*. Characterizing the Dirac cone and identifying band gaps in materials which lift the degeneracy at *K* is one common objective of 2D materials science. However, a very high resolution in reciprocal space is required to obtain an accurate characterization. This highlights one significant limitation of band theory using direct-space orbitals: increasing resolution requires larger supercells, which require more orbitals. Our hybrid mapping ensures the number of qubits required scales only logarithmically with the size of the supercell. Nevertheless, obtaining useful resolutions on multi-dimensional lattices still requires many more qubits than are available on present-day quantum hardware, ensuring that the classical approach to band theory will remain dominant for the time being.

Figure 7c corresponds to a three-dimensional simple cubic lattice. The unit cell consists of a single atom with an *s* orbital and three *p* orbitals. Rest energies and hopping parameters are selected to qualitatively match the electronic band structure of polonium, which forms a simple cubic lattice in standard conditions (on-site Coulomb interaction and relativistic corrections are required for a more accurate representation)^25,26^. Our results use a supercell size of $N=8$ in all three dimensions, requiring nine qubits in the site register and four in the orbital register, for a total of thirteen qubits. Note that the path we have shown through reciprocal space includes two redundant branches, $\Gamma R$ and *XM*. In particular, note that the quantum results for the $R\Gamma$ branch happen to be of much lower quality than those of the $\Gamma R$ branch, despite considering the exact same momentum vectors. This highlights the probabilistic nature of quantum devices, and the urgent need for robust operator estimation and optimation protocols. This need is further exacerbated by the thermal noise and low-fidelity gate operations which plague present-day quantum hardware, and the prevalence of barren plateaus in most cost functions of interest^27^. The purpose of this paper is to showcase the algorithm, rather than obtain high-precision results, and so we have favored simplicity and ease of implementation over rigor. However, the interested reader should be aware that developing efficient and robust quantum protocols is a highly active and relevant area of research, especially in operator estimation^24,28–31^, optimization^32–36^, and error mitigation^37–40^.

## Conclusion

We have presented a novel approach to electronic band structure calculations in a quantum computer by adopting a basis of local atomic orbitals. This has enabled us to prepare a single cost function which can be used for all $\mathbf{k}$ and for all bands. This is an improvement over previous approaches^9,12,13^, which require recalculating new matrix elements for each point in $\mathbf{k}$ space and add additional overlap terms in the cost function to explore higher bands. In exchange, we require an additional $\log N$ qubits, where *N* is the resolution in $\mathbf{k}$ explored by the band structure.

While band structures are obtainable through classical approaches, the surge of quantum algorithms developed in recent months heralds a new era of computational materials science. Quantum computing enables efficient electronic structure calculations of highly correlated systems for which the single-electron approximation fails. The most promising approaches^7,8,10^ assign a unique set of qubits for each periodic basis function, requiring $O(N)$ qubits, although this can be reduced somewhat by tapering methods^10,41^. With further research, we believe the hybrid first/second quantized qubit mapping for periodic systems presented in this paper may be adapted to accommodate multiple electrons, *exponentially* reducing the number of qubits required to express the system. The quantum circuit can be adjusted to express multi-electron states, long-range interactions can be accommodated by introducing entanglement between the orbital and site registers, and additional multi-body terms can be added to the cost function.

One particular difficulty in adapting our approach to multiple electrons is the need to enforce fermionic anticommutation relations, which we have omitted to fully exploit the single-electron approximation. Mapping multi-body fermionic integrals onto the hybrid first/second quantized registers is a subject of further research. Alternatively, one could develop a novel quantum circuit which enforces the appropriate antisymmetry relations on the ansatz directly. Such a strategy is consistent with the theme of this paper: simplifying the cost function and measurement complexity of the VQE algorithm by creatively introducing symmetries and constraints in the quantum circuit.

## Supplementary Information

Supplementary Information.
