# Supplementary material for: Quantum algorithm for electronic band structures with local tight-binding orbitals
Source: Sci Rep. 2022 Jun 14;12:9867. doi: 10.1038/s41598-022-13627-x (PMC9198033; doi:10.1038/s41598-022-13627-x)
Supplement: Supplementary file 1 — Supplementary Information. [file 41598_2022_13627_MOESM1_ESM.pdf]

Supplementary Information for: Quantum algorithm for electronic band structures with local tight-binding orbitals

Kyle Sherbert[1], Anooja Jayaraj[1], and Marco Buongiorno-Nardelli[1,*]

[1]Department of Physics, University of North Texas, Denton, TX 76203, USA.
[*]mbn@unt.edu

ABSTRACT

While the main thrust of quantum computing research in materials science is to accurately measure the classically intractable electron correlation effects due to Coulomb repulsion, designing optimal quantum algorithms for simpler problems with well-understood solutions is a useful tactic to advance our quantum "toolbox". With this in mind, we consider the quantum calculation of a periodic system's single-electron band structure over a path through reciprocal space. Previous efforts have used the Variational Quantum Eigensolver algorithm to solve the energy of each band, which involves numerically optimizing the parameters of a variational quantum circuit to minimize a cost function, constructed as the expectation value of a Hamiltonian operator. Traditionally, a unique Hamiltonian operator is constructed for each k-point, so that many cost functions, each with their own parameter space, must be optimized to generate a single band. Similarly, calculating higher bands than the first has traditionally involved modifying the cost function with additional overlap terms to ensure higher-energy eigenstates are orthogonal to those of lower bands. In this paper, we adopt a direct space approach, using a novel hybrid first/second-quantized qubit mapping which allows us to construct a single Hamiltonian, and a single cost-function, suitable for solving the entire electronic band structure. The k-point and the band index are selected by additional parameters in our quantum circuit, rather than through modifications to the cost function. The result is a technically and conceptually simpler approach to band structure calculations on a quantum computer. Moreover, we expect that the tools developed herein will motivate new strategies for tackling highly-correlated materials beyond the grasp of classical computing.

Commuting Groups in the Site Register

In this appendix, we show that the sum $\sum_\nu |\nu\rangle\langle\nu+1|$ can be decomposed into real and imaginary parts $\hat{A}_{\mathcal{N}}$, $\hat{B}_{\mathcal{N}}$, each consisting of $\Theta(\log N)$ commuting groups.

Let $\nu < N$ be an integer with a binary representation $\nu_{n-1}..\nu_q..\nu_0$, where $n \equiv \log N$. For a specific ν, let r index the least significant qubit q such that $\nu_q = 0$. Defining the substring $\nu^{(r)} \equiv \nu_{n-1}..\nu_{r+1}$ and the projection operator $\hat{\Pi}^{(r)} \equiv (|1\rangle\langle 0|)^{\otimes r}$, the binary relationship between ν and $\nu+1$ yields

$$|\nu\rangle\langle\nu+1| = |\nu^{(r)}\rangle\langle\nu^{(r)}| \otimes |0\rangle\langle 1| \otimes \hat{\Pi}^{(r)}. \tag{1}$$

We are free to rearrange our sum $\sum_{\nu=0}^{N-1} \to \sum_{r=0}^{n-1} \sum_{\nu^{(r)}=0}^{2^{n-r-1}}$, grouping all ν with the same r together. We must also include the term $|N-1\rangle\langle 0| = \hat{\Pi}^{(n)}$, which does not belong to any r class:

$$\sum_{\nu=0}^{N-1} |\nu\rangle\langle\nu+1| = \hat{\Pi}^{(n)} + \sum_{r=0}^{n-1} \sum_{\nu^{(r)}=0}^{2^{n-r-1}} |\nu^{(r)}\rangle\langle\nu^{(r)}| \otimes |0\rangle\langle 1| \otimes \hat{\Pi}^{(r)}. \tag{2}$$

The sum $\sum_{\nu^{(r)}} |\nu^{(r)}\rangle\langle\nu^{(r)}|$ reduces to the identity operator $\hat{I}^{\otimes n-r-1}$. We will omit this factor for conciseness, but note that every term in what follows is padded on the left with $\hat{I}$ operators to form an n-qubit Pauli word.

We now introduce the notation $\{{n \atop k}\}_Y^X$ to represent the *sum* of all n-length Pauli words with $n-k$ $\hat{X}$ operators and k $\hat{Y}$

operators. For example, $\left\{ {3 \atop 2} \right\}_Y^X = \hat{X}\hat{Y}\hat{Y} + \hat{Y}\hat{X}\hat{Y} + \hat{Y}\hat{Y}\hat{X}$. Applying the Pauli mappings for $\left|\nu_q\right\rangle\left\langle\nu_q'\right|$ (Eqs. 15 in the main text):

$$\hat{\Pi}^{(r)} \equiv \hat{A}^{(r)} + i\hat{B}^{(r)}; \tag{3a}$$

$$\hat{A}^{(r)} = \frac{1}{2^r}\sum_k (-1)^k \left\{ {r \atop 2k} \right\}_Y^X; \tag{3b}$$

$$\hat{B}^{(r)} = \frac{1}{2^r}\sum_k (-1)^{k+1} \left\{ {r \atop 2k+1} \right\}_Y^X. \tag{3c}$$

Setting aside the coefficients, $\hat{A}^{(r)}$ includes all r-length Pauli words consisting of $\hat{X}$ operations and an even number of $\hat{Y}$ operations, while $\hat{B}^{(r)}$ includes those with an odd number of $\hat{Y}$ operations. Note that all terms within $\hat{A}^{(r)}$ commute, as do those within $\hat{B}^{(r)}$.

Finally, we write the sum $\sum_\nu |\nu\rangle\langle\nu+1|$ as:

$$\sum_{\nu=0}^{N-1} |\nu\rangle\langle\nu+1| \equiv \hat{A}_{\mathcal{N}} + i\hat{B}_{\mathcal{N}}; \tag{4a}$$

$$\hat{A}_{\mathcal{N}} = \hat{A}^{(n)} + \frac{1}{2}\sum_{r=0}^{n-1}\left(\hat{X}\otimes\hat{A}^{(r)} - \hat{Y}\otimes\hat{B}^{(r)}\right); \tag{4b}$$

$$\hat{B}_{\mathcal{N}} = \hat{B}^{(n)} + \frac{1}{2}\sum_{r=0}^{n-1}\left(\hat{X}\otimes\hat{B}^{(r)} + \hat{Y}\otimes\hat{A}^{(r)}\right). \tag{4c}$$

For fixed r, all Pauli words appearing within each sum commute. Therefore, each operator $\hat{A}_{\mathcal{N}}$, $\hat{B}_{\mathcal{N}}$ contains $\Theta(n)=\Theta(\log N)$ commuting groups. These groups are not *qubit-wise* commutative, and the basis rotation circuit required to transform each Pauli word into the set $\mathcal{Q}_n$ is non-trivial. Nevertheless, Yen et al.[1] provide a procedure to derive the necessary rotation, which we have presented in Fig. 6f of the main text.

References

1. Yen, T. C., Verteletskyi, V. & Izmaylov, A. F. Measuring All Compatible Operators in One Series of Single-Qubit Measurements Using Unitary Transformations, DOI: 10.1021/acs.jctc.0c00008 (2020). 1907.09386.
